# “There’s absolutely no downside to this, I mean, except community opposition:” A qualitative study of the acceptability of vending machines for harm reduction

**DOI:** 10.1186/s12954-023-00747-4

**Published:** 2023-02-28

**Authors:** Rebecca E. Stewart, Nicholas C. Cardamone, Emily Loscalzo, Rachel French, Collin Lovelace, Winna Koe Mowenn, Ali Tarhini, Linden Lalley-Chareczko, Kathleen A. Brady, David S. Mandell

**Affiliations:** 1 University of Pennsylvania Perelman School of Medicine, Philadelphia, Pennsylvania USA; 2 Philadelphia FIGHT Community Health Centers, Philadelphia, Pennsylvania USA; 3 University of Pennsylvania School of Nursing, Philadelphia, PA 19104 USA; 4 Philadelphia Department of Public Health, Philadelphia, Pennsylvania USA

**Keywords:** Harm reduction, Vending machines for harm reduction, Opioid use disorder, HIV

## Abstract

**Background:**

Vending machines for harm reduction (VMHR) are an innovative approach to deliver life-saving materials, information, and treatment for hard-to-reach populations, particularly for persons who inject drugs. The current study explores stakeholders’ perspectives on the feasibility and acceptability of VMHR in Philadelphia.

**Methods:**

From October 2021 to February 2022, we conducted 31 semi-structured interviews with potential end users, staff, and leadership at a local federally qualified health center, and community members. Trained coders extracted themes from interview transcripts across four key domains: materials and logistics, location, access, and community introduction.

**Results:**

Interviewees from all stakeholder groups endorsed using VMHR to provide supplies for wound care, fentanyl test strips, naloxone, and materials to connect individuals to treatment and other services. Dispensing syringes and medications for opioid use disorder were commonly endorsed by health center staff but were more controversial among potential end users. Even within stakeholder groups, views varied with respect to where to locate the machines, but most agreed that the machine should be placed in the highest drug use areas. Across stakeholder groups, interviewees suggested several strategies to introduce and gain community acceptance of VMHR, including community education, one-on-one conversations with community members, and coupling the machine with safe disposal of syringes and information to link individuals to treatment.

**Conclusions:**

Stakeholders were generally receptive to VMHR. The current study findings are consistent with qualitative analyses from outside of the USA and contribute new ideas regarding the anticipated community response and best methods for introducing these machines to a community. With thoughtful planning and design, VMHR could be a feasible and acceptable modality to reduce death and disease transmission associated with the opioid and HIV epidemics in cities like Philadelphia.

**Supplementary information:**

The online version contains supplementary material available at 10.1186/s12954-023-00747-4.

## Background

The opioid overdose epidemic in the USA continues unabated, further exacerbated by the rise of synthetic opioids and the COVID-19 pandemic [15]. Fatal overdoses rose by nearly 40% in the USA from 2019 to 2020, and disproportionately among Black individuals [12, 15]. In response to the COVID-19 pandemic, in March 2020, the U.S. Drug Enforcement Agency relaxed regulations on opioid use disorder treatment  10. This policy change permitted telehealth prescription of controlled substances and medication delivery services through mobile outreach units, which are strategies that continue to engage individuals three years into the pandemic [20, 21, 26, 36].

To alleviate pandemic-related treatment disruptions, public health officials also called for an expanded investment in harm reduction, an approach that emphasizes pragmatic strategies to help individuals mitigate the risk associated with their substance use and increase treatment readiness [19, 23]. These strategies include education about safe drug use, providing safe injection and safe sex supplies, syringe exchange programs, supervised injection facilities, and opioid agonist therapy [4, 31]. Harm reduction strategies improve healthcare access for people who use drugs, reduce fatal drug overdoses, and mitigate the transmission of diseases such as human immunodeficiency virus (HIV) and hepatitis C [11, 31]. The harm reduction model has received more support in recent years in the US and has been integrated in a number of federal, state, and local health policies, such as allowing states to purchase naloxone with federal funds, statewide protections for pharmacists who dispense naloxone, and city-led syringe exchange programs [6, 9, 25].

A promising and innovative harm reduction intervention that has emerged in the last fifteen years is the vending machine for harm reduction (VMHR). VMHR is an umbrella term for a range of mechanized devices that deliver materials, information, and treatment to historically excluded populations, particularly for people who inject drugs (PWID) [24, 35]. Although the earliest forms of VMHR were designed to collect and distribute syringes, some machines also dispense other harm reduction materials, such as naloxone, fentanyl test strips, medications for opioid use disorder (MOUD, i.e., buprenorphine, methadone, and naltrexone) and safe supplies of pharmaceutical opioids [13],  [27, 39]. VMHR that dispense sterile injecting supplies have been implemented in Australia, Taiwan, Mexico, Canada, and a number of European countries [14, 16, 27–29]. A review of VMHR in Europe and Australia find that they commonly reached high-risk and marginalized populations, including people experiencing homelessness, women, and disenfranchised racial and ethnic groups [16]. When deployed in jails in Switzerland and Germany, VMHR increased access to sterile injecting equipment without increasing drug use frequency and were perceived as most acceptable when the utilizer could maintain anonymity [16].

Despite their promise, VMHR can introduce many multifaceted and contradictory feasibility and acceptability concerns. Stakeholders in qualitative studies of VMHR consider a number of practical questions, such as where the machines would be placed, how to keep the machines stocked, what supplies the machines would dispense, and whether the items should be free or have a cost [17, 27]. For instance, a survey of PWID in Tbilisi, Georgia found that 42% of those who would be willing to use a VMHR rated the availability of free supplies as the most important reason to use the machine [28]. In addition, community stakeholders commonly raise concerns about the misuse and vandalism of machines, especially if individuals could freely access items [17]. Although these concerns could be remedied through increased security, monitoring, and time restrictions, additional requirements may take away from the anonymity and convenience that make VMHR attractive to PWID [17, 16].

The lack of acceptability in different communities may obstruct initial or sustained implementation of VMHR, which is concerning given that attitudes toward VHMR can vary widely across stakeholder groups [17, 28–30]. For example, community members in Tijuana, Mexico, a candidate location for a VMHR program, were split between those that saw it as an important means of harm reduction and those who opposed it based on beliefs that the supplies provided in VMHR would enable drug use [29]. Those who argue against VMHR have emphasized the potential for VMHR to promote drug use, increase crime, and spread drug related litter [17, 28–30], despite a lack of evidence to support these claims [7, 8].

In the USA and Canada, there are VMHR available for rental or purchase that are already in use in several jurisdictions. They range from providing only one item (e.g., naloxone) to providing a number of items, including safe injection kits, fentanyl test strips, and wound care supplies. Vancouver, Canada, deployed a number of “MySafe” machines which use biometric scanning to dispense safe supplies of opioids to prescription holders [39]. Although published evaluations of VMHR have almost exclusively assessed VMHR sites in Europe and Asia, preliminary evidence from pilot VMHR in the US show promising results. In Cincinnati, Ohio, a syringe service program offers safe injection kits (excluding syringes), safer smoking kits, naloxone, and personal protective equipment, through an outdoor VMHR that can be accessed 24/7 with a personal code (“Safer-Use Supplies,” n.d.). A recent evaluation finds that the introduction of this VMHR site dramatically increased the accessability of harm reduction products and services to PWID, even among those who had never reportedly used harm reduction services. Although causality cannot be established, there were fewer unintentional overdoses and new HIV diagnoses in the county in the year following the VMHR’s implementation [3]. The public health department of Las Vegas, Nevada, deployed six VMHR in 2019, which hold syringes, naloxone, pregnancy tests, safe sex kits, personal hygiene kits, first aid kits, and sharps containers. Similar to the results from the VMHR in Cincinatti, preliminary evidence from this site shows that rates of naloxone dispensation from these machines were associated with subsequent reductions in overdose fatalities [2]. In 2022, Philadelphia introduced “Narcan Near Me” towers, which dispense free naloxone. Naloxone dispensing machines have also been deployed in other areas of Pennsylvania, Wisconsin, Kentucky, and Rhode Island, due in part to the expansion of naloxone access laws [33].

The objective of the present study was to ascertain the acceptability and feasibility, among different stakeholder groups, of implementing VMHR in Philadelphia. We endeavored to identify barriers that would inform where VMHR should be placed and what harm reduction materials they should contain. This exploratory, multi-stakeholder qualitative inquiry serves as a step toward establishing additional and expanded VMHR in Philadelphia. The study also focuses on contributing to the scientific literature on VMHR and gaining rapid and actionable knowledge about their use, providing a valuable service to our community and public health partners.

## Methods

### Participants

The University of Pennsylvania and City of Philadelphia Institutional Review Boards approved all procedures. We identified three stakeholder groups to recruit for study participation: (1) end users: individuals served by a Federally Qualified Health Center (FQHC) who have a history of injection drug use and could be potential VMHR users; (2) FQHC staff: patient-facing personnel and administrative leadership and (3) a diverse community stakeholder group that included residents, clergy members, members of community advisory boards and neighborhood organizations. We recruited potential participants from October 2021 to February 2022 through multiple methods including flyer distribution, electronic letters, social media posts, and word of mouth. Interviews were conducted via phone or virtual meeting. Participants were offered a $25 Amazon gift card for participation.

### Interview

In consultation with a local FQHC and the Philadelphia Department of Public Health, we designed a semi-structured interview to address the feasibility and acceptability of VMHR. Working from scripts and an outline, standard definitions of harm reduction and VMHR were provided. The interviewer listed potential materials that VMHRs could dispense: safe disposal and injection kits, fentanyl test sets, items for wound care, naloxone, condoms, and HIV self-tests. Initial questions were open-ended and gave the interviewee an opportunity to describe what they knew about harm reduction, their initial thoughts about a VMHR, and list materials they would like to see in a VMHR, regardless of whether they were relevant to harm reduction. The interviewer then asked about possible challenges to implementing VMHR and how best to introduce VMHR to the community, with specific probes about acceptance, privacy, alternative materials, logistics, and legality. For example, those that may be the potential end users of VMHR were asked how the machine could best serve them, whereas the FQHC staff and leadership interviews focused on the clinical and practical implications of implementing VMHR in their organization. Community members were asked about the potential acceptability of VMHR in their neighborhood and how to introduce the machines to their community. Interviews were digitally recorded, professionally transcribed, anonymized, and lasted from 22-46 minutes. The interview guide is available in Additional file 1.

### Analysis

Analysis was guided by modified grounded theory, which provides an adaptive approach to collecting and analyzing qualitative data and has produced robust theoretical models of social behavior in healthcare settings (Damschroder et al. 2009). Following the process outlined by Charmaz and Belgrave [5], we first conducted iterative open coding on sets of transcripts to identify initial themes, then used the most significant codes to synthesize the total set of transcripts [5]. We held three exploratory coding sessions with all interviewers, coders, and our partners at the FQHC. For each session, attendees read two transcripts and identified emergent candidate codes. The lists were combined and condensed into a joint list of four domains which were developed into a coding manual. The manual was tested and refined over eight additional transcripts until two independent coders (RF, CL) achieved a kappa of at least 0.60 with the principal coder (NC). Coders (RF, CL, NC) categorized question and response chunks from assigned transcripts using the qualitative analysis software, NVivo, and met routinely to discuss example cases. Three members of the authorship team (RS, NC, and CL) conducted an additional analysis to identify the prevalence of themes by stakeholder group. When apparent, we compare and report differences by stakeholder group below.

## Results

### Sample characteristics

We interviewed 31 individuals across the four stakeholder groups. The eight potential end users included individuals living with HIV and/or viral hepatitis, individuals who reported current or past injection drug use, and unhoused individuals. From the FQHC, we interviewed nine staff (e.g., social workers, prison linkage specialists, nurse managers, and behavioral health consultants) and six leaders (e.g., chief executive officer, legal officer, and quality assurance officer). The eight community members comprised a diverse group and included individuals from multiple zip codes in Philadelphia County. We held interviews with three community advisory board members, two leaders of a faith-based organization, and three residents. Self-reported demographic characteristics are provided in Table 1.

**Table 1:** Demographic characteristics

	Total (*N*=31)
Gender	
Male	48.3%
Female	45.2%
Transgender	6.5%
Age at interview (years)	47.26
Ethnicity	
Black	48.1%
White	44.4%
Multiethnic	7.4%
Hispanic	10.7%
Highest level of education	
Less than high school diploma	3.6%
High school diploma	21.4%
Some college	14.3%
Bachelors	21.4%
Higher than Bachelors	39.3%

### Materials

Every participant received a standard definition of VMHR “as a machine containing harm reduction materials, such as safe disposal and injection kits, fentanyl test strips, items for wound care, naloxone, condoms, and HIV-self tests.” Stakeholders provided their opinion on these example items and proposed additional items that they believed should be housed in a VMHR. In total, suggested materials spanned five broad categories: (1) supplies to reduce drug use harm (e.g., safe smoking supplies, cotton balls, and saline), (2) supplies to reduce risk of viral and disease transmission (e.g., Hepatitis C tests, pre-exposure prophylaxis, condoms, COVID tests, personal protective equipment, masks, and sanitization), (3) family planning and feminine products (e.g., birth control, pregnancy tests, emergency contraception, and menstrual products), (4) general medicine and first aid (e.g., band aids, gauze, hot compresses, oral hygiene kits, medications, emergency tourniquets, defibrillators, and epinephrine) and (5) informational materials (e.g., pamphlets and cards) to connect people to services (e.g., substance use treatment centers, suicide hotlines, crisis centers, homeless shelters, and food pantries) or education about substance use disorders, intimate partner violence, and how to use dispensed materials (e.g., how to administer naloxone or conduct a HIV self-test).

Stakeholders were generally positive about the idea of a machine that dispensed harm reduction materials and could potentially save lives. Naloxone garnered universal support and was also seen as acceptable to others. Stakeholders were enthusiastic about wound care and first aid equipment. Participants in all stakeholder groups mentioned the importance and benefit of information materials. As one community member stated:

I would also assume we would want to stuff prevention messages into anything … You know, a little card like, ‘Need help? This is who you call.” …something little, but you know you don’t ever want to miss an opportunity.

Similarly, an FQHC staff member shared:

The machines themselves sort of share information with patients, right? So, like I would love that even if you’re giving them a needle, you’re also giving them a ‘Here’s some Suboxone places.’

Fentanyl test strips were also viewed positively across stakeholder groups. One end user said that the inclusion of fentanyl test strips and naloxone, “would be a really positive thing,” for his area which had seen a recent increase in opioid use.

In contrast, interviewees held conflicting views on the feasibility and acceptability of including safe injection or smoking kits, clean needles, and MOUD. Syringes proved to be the most controversial. In general, FQHC members offered the greatest support, with their chief concern being proper disposal. Community members also supported the provision of clean syringes and saw VMHR as an opportunity to deliver aspects of a safe injection site or syringe exchange site in another, more discreet form. As one community member described:

Like, we know syringe sites work, we know that. So, I think the next best thing because you can’t really have them would be a vending machine where it’s accessible for people. Like if it’s there, people will take it.

Individuals in the end user group similarly demonstrated some support but included a number of interviewees that were against the inclusion of clean needles because they thought it would promote drug use and lead to more litter. One end user expressed reluctance:

Uh… [I would] not really [be okay with needles being the machine]. I have to be honest- nah, because that’s just giving them a way to get high. It’s easy access because instead of going to the store, and they’re [going to be] selling them or stealing them...

Opinions on MOUDs were divided within stakeholder groups. One FQHC staff member was in favor of a machine that contains Suboxone (buprenorphine and naloxone) to prevent relapse in emergency situations when an individual’s dose is lost. However, another FQHC staff member noted the complexity of providing such a controlled medication through a machine:

Yeah, providing Suboxone would present a lot of challenges in terms of safety and making sure that folks don’t prioritize vandalizing the machines in order to steal the Suboxone. You probably would deal with issues concerning the [Drug Enforcement Agency].

### Location

Participants discussed the location of the machine at length, including views of desirable or undesirable geographic areas. Almost all interviewees, regardless of stakeholder type, suggested Kensington (an area in Philadelphia with the greatest drug activity), as the most appropriate location for the machine. For example, one community member explained: “Where are they going to make the most impact? Where are they going to meet the most people who are doing this? I would say is obviously Kensington.” However, a number of participants suggested areas outside of Kensington to target neighborhoods with less visible drug use and provide harm reduction materials to underserved communities. One community member advocated for an intentionally conspicuous placement to potentially reduce stigma and normalize the presence of VMHR:

That’s why I said if they’re put in areas where you would not expect to see them – City Hall [for example] – I think then people aren’t going to mind seeing them pop up in areas where they’re needed also.

Despite the potential advantages of the machines being visible, many interviewees agreed that this should be balanced with protecting the user’s privacy and confidentiality. Nevertheless, many participants expressed that privacy concerns would be fewer in areas with visible open air drug use, like Kensington. One end user was not concerned for user privacy because, “they’re out on the streets where I’m from – I’m from Kensington – they’re out on the street, they don’t care.”

Participants also discussed where the machines should be placed in relation to existing buildings and landmarks. Across the three stakeholder groups, stakeholders recommended a variety of places, including public transit infrastructure, recovery centers, libraries, gas stations, grocery stores, schools, and police stations. Stakeholders most commonly endorsed placing VMHR in and around subway stations, particularly in stations close to Kensington. FQHC leadership and staff were generally receptive to the idea of operating a VMHR at their center. However, both administrators and clinicians raised a variety of concerns related to how VMHR would affect patient care, privacy, and willingness to access the FQHC. Although FQHC staff recognized the potential for VMHR to serve individuals at all hours, at least one staff member thought that VMHR may be more helpful in other locations given that the FQHC already provides harm reduction materials directly.

Most stakeholders addressed the pros and cons of different placements. Stakeholders viewed an attractive location for VMHR as one that balanced visibility, security, and privacy. Visibility was seen as increasing security and accessibility, and reducing stigma, but was potentially concerning due to end user’s privacy concerns and community opposition. In general, FQHC staff were more sympathetic to privacy concerns than the other groups. For instance, one staff member conceded that individuals may be more comfortable accessing fentanyl strips through a machine than asking their treatment provider. Yet, other FQHC staff were concerned that their patients would access the machine in lieu of seeing a treatment provider, reducing clinician and patient interaction and engagement. Interviewees discussed the nuances of placing the machine in other settings, such as whether it was prudent to place it in a waiting room given the lack of confidentiality or a bathroom given the lack of supervision. As one end user described,

[VMHR] being in the bathroom might encourage [drug] use in the bathroom as well. So, they may not want to do it in the bathroom, but it has to be in a place where people don’t feel like everybody’s watching them take items from the machine or get items dispensed from the machine.

Finally, some interviewees suggested placements that would preserve 24/7 access to VMHR as well as decrease vandalism and theft. For example, several interviewees suggested small, kiosk-like housing, comparable to banks that have ATMs in their entryways.

### Access

Stakeholders mentioned a variety of considerations for how the machine should be accessed and under what conditions. There was disagreement about whether items should be free. FQHC staff often noted that cost could be prohibitive for many individuals who could be served by the machine:

How would someone pay since this is a marginalized community we’re talking about, and income is very low, how would they be able to afford to get something out of those machines?

Several individuals with lived experience pointed out that some items (such as syringes) can be obtained freely elsewhere so, “they're going to go where they can get them for free.” On the other hand, many participants reported misgivings about free items, commonly expressing concerns that the machine would be “cleaned out”. One community member explained:

…the way I look at it- if everything in it is ‘free,’ I really think that one person’s going to go back because, ‘they will let me take 10 of these or 12 of these’ or keep taking them until there’s none left.

There were also ideological concerns about the optics of free items as “condoning drug use.” As one end user cautioned:

if the access to the materials they’re contained in is free, then I would almost think you could legally view that as an inducement to get high.

Many participants offered alternative methods of accessing items, such as a machine-specific access card, allowing access through a Medicare or SNAP benefits card, non-money token, or user-specific codes.

Some interviewees recommended imposing more conditions of access to have more control over the machine’s users, such as restricting access to children, limiting access to certain times of day, or surveilling users more closely. Yet, stakeholders expressed competing concerns of balancing surveillance for security with privacy and accessibility for users (particularly for machines outside of highly visible drug activity areas). Stakeholders brought up related concerns that a machine would attract law enforcement that would turn potential users away or criminalize users for accessing or obtaining drug paraphernalia. Some FQHC staff members recognized the clinical utility of collecting data on what sort of materials were being accessed. One staff member mused:

I would say it would have to be somewhere linked to us and that someone would actually have to monitor how many times that person has used the vending machine within a day, within week and what exactly that they’re, you know getting to, right?

Some interviewees acknowledged that the accessibility and anonymity of the machine may depend on the materials. All stakeholders who discussed naloxone agreed that it should be dispensed freely and quickly in case of an emergency.

### Community

We asked stakeholders about the community response to VMHR and how to improve community acceptance. Interviewees generally assumed that there would be dialog between the implementers of the machine and the community in which they would be placed. Stakeholders offered a variety of strategies toward how best to engage the community around VMHR, including group-based or one-on-one educational meetings and open forums addressing concerns, relationship building with neighborhood leaders or community organizations, and advertising through flyer distribution and social media. Stakeholders highlighted that coupling VMHR with safe and increased disposal of needles would be a particularly important selling point to the community. A number of interviewees recommended starting with less stigmatized materials (e.g., water, snacks, naloxone, and information on substance use treatment) and gradually introducing more controversial materials (e.g., condoms, safe smoking kits, and syringes). Other features that would enhance acceptability of machines include limiting children’s access, and VMHR attendants such as peer specialists or guards to interface, educate, and assist. FQHC employees and community stakeholders promoted education around harm reduction efforts in general, to help the community to understand, “that ultimately the community is protected through these measures.”

Interviewees suggested reasons why some community members might be against the machines. One explanation was that the VMHR (providing items such as safe injection kits) condones and encourages drug use. End users were the only stakeholder group to note that they themselves held this belief. One end user in recovery from OUD described his opposition to syringes:

Because I’m a recovering heroin addict and I feel like if it was easier for me to get syringes and things like of that nature, then I might not have stopped using drugs.

Another proposed reason for community opposition was what the machine signified about the neighborhood and that the machine might attract increased drug activity, drug litter, and drug-using individuals. As one community member explained:

If I’m driving down the street and I see one of those machines, does that immediately label that neighborhood as a more drug-prone neighborhood?

Participants from all stakeholder groups drew comparisons with the city’s recent initiative to introduce safe injection sites and reflected on lessons learned. As one FQHC employee noted:

I think in different neighborhoods where there was discussion about potentially putting safe injection facilities, there was a lot of uproar and so, I don’t know whether you would get the same uproar putting something like a vending machine in the same areas.

Many stakeholders believed the main lesson learned from the failed safe injection site initiative was a lack of communication between the implementers and the community. Despite this, most stakeholders were hopeful that with a thoughtful introduction coupled with significant community outreach, discourse, and education, the community would be receptive to VMHR due to the salience and prevalence of drug use in many neighborhoods. Some community members highlighted that the need for VMHR was pressing given the severity of the opioid overdose epidemic and that universal community acceptance was neither necessary nor possible. As one community member described:

We have to start thinking radically about what to do with this crisis…we definitely need some radical ideas and folks need to embrace that we need some radical ideas to stop people from dying.

## Discussion

Our study identifies critical decision points relating to the feasibility and acceptability of VMHR in Philadelphia. There was striking consensus across many domains among our stakeholder groups. For example, there was universal agreement on providing naloxone, wound care and first aid materials, and informational materials to connect individuals to services and treatment. The agreement about these harm reduction materials may indicate that public attitudes are shifting in favor of certain harm reduction interventions for PWID. No stakeholders mentioned a moral hazard of providing naloxone or provided other ideological debates that naloxone promotes or encourages drug us [34, 38]. Stakeholders proposed a wide breadth of materials that extend beyond a VMHR’s mission to address the needs of opioid users and reduce harm from wounds and viral infection. The large number of proposed items speaks to the great need in the community and that VMHR may have the capacity to address these needs.

Consistent with previous research, there was less agreement among stakeholder groups about providing syringes or clean needles, with end users holding the most negative views. A central finding of our study was the perception that VMHR or certain materials (e.g., needles, safe injection kits, and safe smoking kits) promote or encourage drug use. While end users were the only group to personally subscribe to this idea, all stakeholder groups noted the stigma against harm reduction interventions as a primary reason for community opposition. This is a critical opportunity for broad public health messaging about harm reduction, as well as for harm reductionists to learn how to frame harm reduction in ways that are persuasive to the community and particularly to people who use or inject drugs. There is a body of research that contradicts that harm reduction interventions promote substance use. This evidence supports the reverse, that harm reduction interventions have been linked to positive health outcomes for PWID both in the short term, and in the long term by preventing infectious diseases and connecting people to treatment [11, 22, 31], This evidence is necessary but not sufficient to combat misinformation about harm reduction. Future research should test and compare messaging packages that effectively combat the idea that “you are just helping them get high.” There have been promising professional education efforts to reduce provider stigma toward harm reduction, and related efforts are needed toward educating the general public that harm reduction is effective, a better option for many opioid users who are not currently interested in treatment, and better for the community in both the short and long term [37]. Before any VMHR implementation, any ambassadors of VMHR should be provided with coaching, evidence-based toolkits, and talking points about harm reduction and in response to its most common critiques to encourage community buy-in.

Participant stakeholders had many suggestions for increasing community and neighborhood acceptance of VMHR. While we directly asked how to enhance community acceptance of VMHR, Philadelphia’s recent failure to implement a safe injection facility following intense community backlash meant that this topic was on participants’ minds. Many stakeholders compared the approach of the safe injection site introduction and provided different strategies for introducing a VMHR. Education and small community meetings were the most recommended strategies. Stakeholders agree that these machines should be placed in areas of highest drug use and need. Our findings also indicate that a VMHR becomes a symbol or a marker for what and who a community is. For example, a number of community members and end users believed that a VMHR would identify a neighborhood as one with illicit drug use [8], or even attract and promote more drug use. Some stakeholders provided alternative compelling arguments for placing machines everywhere or in highly visible locations to reduce stigma and increase acceptance.

Stakeholders converged on a common set of ideas to make the machine more acceptable: link it to increased needle disposal in the neighborhood and include informational materials connecting people to services and treatment. There are many research and evaluation opportunities to test if or what kind of messaging effectively connects individuals to treatment or other harm reduction services. One proximal impact of the informational materials is that their inclusion increases community enthusiasm for the VMHR. The present findings suggest that VMHR could be made more palatable by including these materials. In general, stakeholders suggested a variety of “foot-in-the-door” approaches to introducing VMHR to the community, which is promising given a VMHR that dispenses naloxone is already in operation in Philadelphia [1, 40].These findings will continue to inform the Philadelphia Department of Public Health and other community sites as they pilot a series of VMHR in waiting rooms in Philadelphia prisons. Based on these findings, the authors have also provide recommendations on community engagement strategies to help reduce community opposition.

### Limitations

Several study limitations should be noted. First, the sample represents a regionally confined subset of stakeholders who responded to a recruitment letter about a VMHR. It is possible that individuals who agreed to participate are not representative of the larger Philadelphia population and that a more diverse sample would show different outcomes. Second, we did not interview any legal professionals. Although several individuals mentioned critical legalities of providing certain materials (e.g., naloxone, fentanyl test strips, needles, suboxone, and no identifiable themes emerged). Future inquiries should seek to understand the multi-level interactions between local, state, and federal laws.

### Conclusions

A suite of public health interventions is necessary to respond to the complex interaction of the opioid and HIV epidemics, the ongoing COVID-19 pandemic, and the unique social and political contexts of diverse urban environments. This study is one of the first to investigate the potential feasibility and acceptability of an innovative modality for delivering harm reduction materials in the USA. Stakeholders were generally positive about a VMHR and provided a number of suggestions to increase community acceptance around this strategy. Given the growing number of VMHR in the USA, more research and evaluation are needed to examine community acceptance of VMHR, and evaluate VMHR effectiveness in increasing access and acceptability of harm reduction supplies. Further, it will be important to learn if VMHR could successfully link individuals to treatment and to explore local potential negative effects of VMHR (e.g., increased litter, increased propensity to use drugs, etc.) that were suggested by stakeholders.

## Supplementary information

**Additional file 1**. Interview script and question guide.

## Data Availability

The datasets used and/or analyzed during the current study are available from the corresponding author on reasonable request.

## Abbreviations

VMHR: Vending machine for harm reduction
MOUD: Medications for opioid use disorder
FQHC: Federally qualified health center
HIV: Human immunodeficiency Virus
