# Supplementary material for: “There’s absolutely no downside to this, I mean, except community opposition:” A qualitative study of the acceptability of vending machines for harm reduction
Source: Harm Reduct J. 2023 Feb 28;20:25. doi: 10.1186/s12954-023-00747-4 (PMC9971672; doi:10.1186/s12954-023-00747-4)
Supplement: Supplementary file 1 — Additional file 1. Interview script and question guide. [file 12954_2023_747_MOESM1_ESM.docx]

Appendix**.** Interview script and question guide

**Participant ID#:**__________ **Interviewer Initials: __**__**____** **Date:** ____________

**Stakeholder Group**

| **End users** | **FQHC staff** | **FQHC administrator** | **Community** |
| --- | --- | --- | --- |

**Introduction:**

Hello, thank you so much for agreeing to talk to us. I’d like to discuss the idea of vending machines for harm reduction which may be introduced in Philadelphia sometime soon. Have you heard of these? There are many potential aspects of this idea that we would like to address, for example, whether this machine and certain materials it will contain are acceptable and feasible in Philadelphia. We are meeting with a few dozen people in the community, such as yourself, who have a stake in this issue to understand what the potential introduction of these vending machines would be like.

I’d like to take this opportunity to remind you that any information you share today is confidential. Your comments will not be linked to you. I will create a report that will summarize, in total, the comments made by all individuals I interview for this project. Before we start, I want to remind you that there are no right or wrong answers to any questions and to please ask me about anything that might be unclear.

Are you in a place private place where you feel comfortable conducting this interview? (If no, wait until they have changed locations or reschedule the interview for another time.)

Do you have any questions before we begin?

**Opening Questions**

1. **First, I’d like to ask you a few general questions about harm reduction. What comes to mind when you hear the phrase harm reduction?**

*Clarify:* By harm reduction, we mean prevention-focused practical strategies aimed at reducing the negative consequences of drug use. Although harm reduction is not a treatment strategy per se, harm reduction interventions can be potentially lifesaving in the moment.

*In our case, we refer to harm reduction as prevention-focused ways at reducing harm caused by drug use.*

*Probe:* I know this can be a divisive issue: may I ask you for a little bit more education from you on your (statement/opinion/ stance)?

*Clarify before going on to next question*: In this case, a vending machine could dispense critical supplies, such as safe disposal and injection kits, fentanyl test sets, items for wound care, emergency medications to prevent opioid overdoses, condoms, and HIV self-tests.

1. **What are your initial thoughts about a vending machine like I described?**

*Ensure participant has clear understanding of potential vending machine, review as necessary*

*Probe (for FQHC staff / administrators & end users):* What about a vending machine at *your* center?

*Probe (for community members):* What about a vending machine in your community?

*Follow up probe for all*: How would a machine like this affect your life?

1. **What might be the greatest challenges / the major factors to consider before introducing a vending machine like the one we have discussed?**
2. **What do you think would be the best way to introduce the vending machine in your organization/community?**
3. **Can you envision other services that would better deliver prevention supplies to those in need?**

**Additional Topics to Address**

1. **Acceptance: What would make a vending machine more acceptable to you?**
   1. **What would make a vending machine more acceptable to the community?**
   2. **How might we introduce this to the community?**
2. **Privacy concerns: Tell me more about privacy concerns.**
   1. **How can we best address privacy concerns for users of the machine?**
   2. **What barriers do you foresee?**
3. **Materials: What materials would you like to see in a vending machine?**
   1. **What materials do you think would be more or less acceptable in a vending machine?**
4. **Logistics: How would you like individuals to access the machine?**
   1. **Where would the machine be located?**
5. **Legality: (***This question is specific to staff or community legal experts. The interviewer may note legal concerns raise by any/all interviewees if spontaneously provided)* **Tell me more about legal barriers to implementing a vending machine for harm reduction.**

**Participant Background**

1. **Background Information (You may refuse to answer any of these questions).**
   1. Please tell me your gender.
   2. Do you identify as Hispanic/Latino?
   3. Please describe your ethnicity/race.
   4. Please tell me your age.
   5. What is your highest level of education? Bachelors (e.g., BA, BSN), masters (e.g., MA, MSW, MFT), doctoral (e.g., PhD, PsyD, MD), or other?

INTERVIEWER COMMENTS*

- 1. Respondent’s level of interest and involvement in answering questions.

1 2 3 4 5

Very low Low Neutral High Very High

- 1. Please estimate the respondent’s understanding of the issues discussed in the interview.

1 2 3 4 5

Limited Partial Average Majority Complete

- 1. Please rate your impression of the knowledge of the respondent in the topic being addressed in this interview.

1 2 3 4 5

Very low Low Neutral High Very High

- 1. Describe any discrepancies, gaps, or other problems with the interview.
  2. Describe any circumstances that occurred while the interview was in progress that may have affected the quality of the interview (i.e., interruptions)?
